# Nutritional Composition and Functional Properties of Commonly Consumed Lentils: Impact of Commercial Lentil Types and Cooking Processes

**DOI:** 10.3390/foods15162898

**Published:** 2026-08-19

**Authors:** Huawei Zeng, Bryan D. Safratowich, Zhenhua Liu, Derek D. Bussan

**Affiliations:** 1 USDA-ARS Grand Forks Human Nutrition Research Center, Grand Forks, ND 58203, USA; 2 School of Public Health and Health Sciences, University of Massachusetts, Amherst, MA 01003, USA

**Keywords:** fiber, lentils, mineral, nutritional composition, resistant starch

## Abstract

**Background/Objectives**: Lentils are part of the legume family, containing high proteins, minerals, fibers, and bioactive compounds. Although lentils are regarded as a functional food due to their health-promoting nutritional profile, the impacts of cultivar differences and cooking processes on their nutritional and functional properties are not fully understood. **Methods**: The nutrition composition (e.g., minerals) and water- or oil-holding capacity (WHC or OHC) of six major raw and cooked lentil types (red, yellow, green, brown, French green, black) were determined by inductively coupled plasma mass spectrometry, as well as biochemical and biophysical assays. **Results**: The mineral, protein, fat, resistant starch, fiber (soluble and insoluble), and total phenolic content of raw lentils are significantly different across types. For instance, iron ranged from 4.6 mg/100 g to 6.8 mg/100 g and protein from 25% to 29%, with the highest levels observed in black lentils. Importantly, the cooking process was a primary factor determining the lentils’ final nutritional composition and functional properties. Compared to raw samples, cooked lentils exhibited marked increases in total dietary fiber (e.g., 38% in red lentils), resistant starch (e.g., 93% in red lentils), phenolic content (e.g., 110% in French green lentils), and oil-holding capacity (e.g., 52% in brown lentils). **Conclusions**: While the nutritional profiles of raw lentils vary significantly between types, their respective WHC and OHC remain relatively stable, and cooking processes enhance nutritional scoring metrics, such as increasing OHC and resistant starch. These data, particularly the high health indices of black lentils, highlight the nutritional value and health benefits of lentil types.

## 1. Introduction

Pulses, nutrient-dense foods, are included in dietary recommendations and several nutritional therapy guidelines [1]. Specifically, lentils (*Lens culinaris*) are a healthy food consumed worldwide due to their high protein, fiber, vitamins, and minerals, such as iron and potassium [2]. In addition to their nutritional value, lentils provide environmental benefits related to their ability to convert atmospheric nitrogen into plant-usable nutrients [3]. Thus, diversifying crop rotations with lentils is a green approach to enhancing soil health and ecosystem productivity [4]. The major lentil types are available in six primary colors (red, yellow, green, brown, French green, and black) depending on the cultivar, seed coat and cotyledon composition [5]. These differences in composition contribute to varying levels of nutrients.

However, lentil consumption is often low in Western diets and has only recently gained popularity in the U.S. [6]. This trend may be attributable to a lack of in-depth knowledge regarding the nutritional and chemical properties of lentils. Furthermore, the impact of thermal treatment, including cooking processes, on lentil nutritional composition remains to be fully determined [7]. Lentils are high in carbohydrates (>30%) including resistant starch, soluble, and insoluble fiber [6]. Cooking processes effectively break down antinutritional factors, such as phytic acid and trypsin inhibitors, enhancing nutrient absorption [8]. However, these methods also alter the nutritional profile, causing variations in dietary fiber, protein and bioactive content [9]. Lentils are small and thin-skinned, and boiling water penetrates them quickly. Boiling lentils at atmospheric pressure is widely considered the most common cooking method because it is efficient and low-maintenance and effectively tenderizes them without a pre-soaking step [10]. The nutritional and functional value of food is affected by ingredient selection and nutrient loss during the cooking process [10]. Consequently, researchers are increasingly interested in the impact of cooking methods on the nutritional, functional, and sensory qualities of legumes [11]. While studies have evaluated the effects of cooking on the nutrients of specific lentil types [12], scant research has simultaneously examined the impact of the cooking process on the nutritional profiles and functional properties of six major lentil types.

Red and yellow lentils lose their hulls during post-harvest processing, while green, brown, French green, and black lentils retain them due to structural differences. Therefore, only dehulled red and yellow lentils and whole green, brown, French green, and black lentils (with hulls) are commonly available for human consumption. This report focuses on commercially available lentil types to reflect daily human consumption. A nutritional comparison of these six lentil types may provide a snapshot of general lentil nutrient intake. We hypothesize that the cooking (via boiling) process increases dietary fiber content and improves functional properties, with differences in the nutritional profile arising from the specific lentil type. This study examined the effects of the cooking process and lentil type on nutritional composition and functional potential, such as water/oil absorption capacity.

## 2. Materials and Methods

### 2.1. Lentils

**Figure 1:**
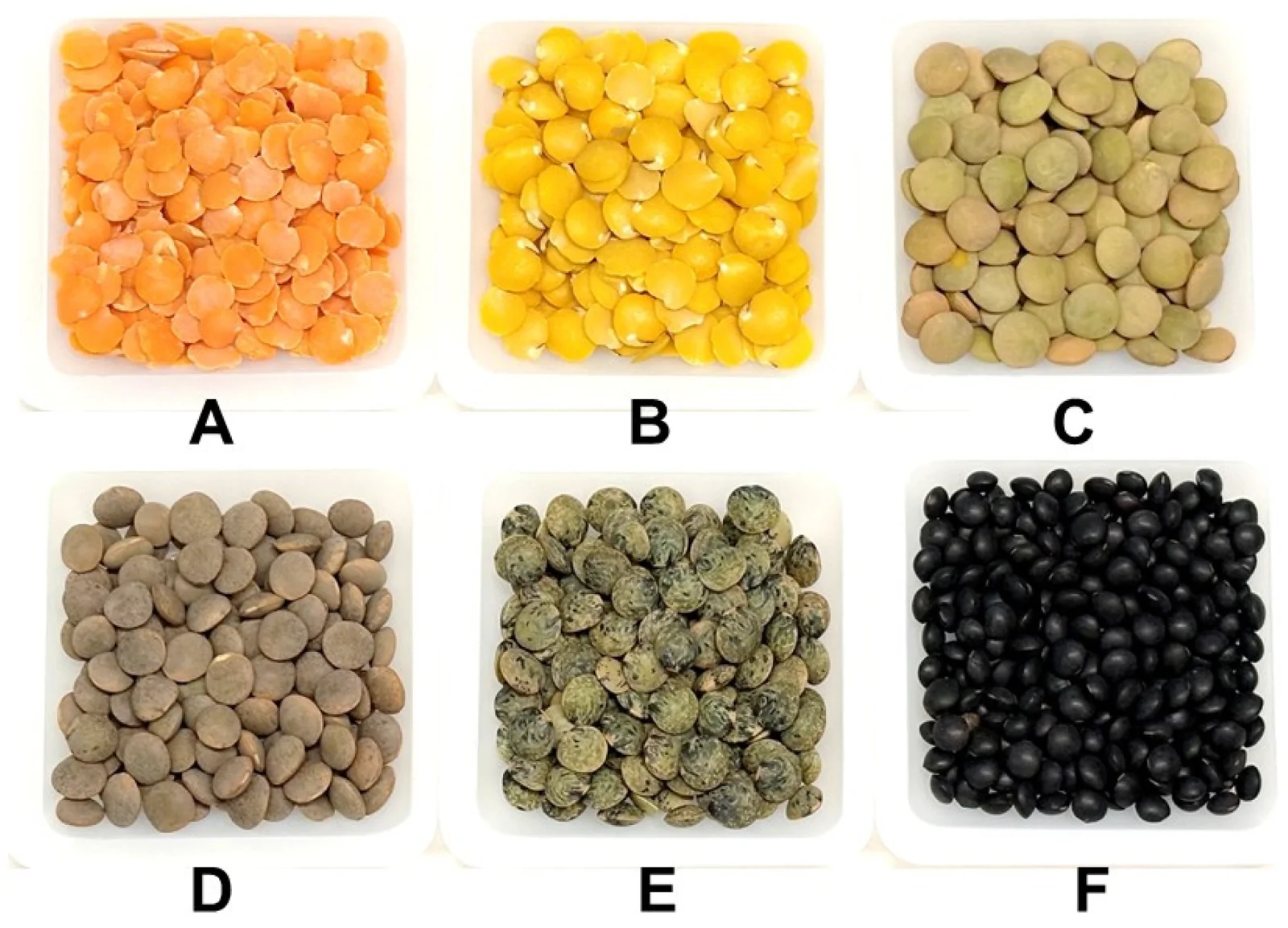
Representative images of (**A**) red, (**B**) yellow, (**C**) green, (**D**) brown, (**E**) French green, and (**F**) black lentils.

Six major lentil types (Figure 1) were sourced from different locations across the U.S. for this study: (1) yellow (lot# B0574), green (lot# A0563 and B0575), and black (lot# B0579) lentils were obtained from Montana state producers via www.clearcreekfoods.com(accessed on 10 April 2024); (2) red (lot# C0585) lentils were obtained from Idaho state producers via www.clearcreekfoods.com (accessed on 10 April 2024); (3) brown (lot# C0584) lentils were obtained from Washington state producers via www.palousebrand.com (accessed on 10 April 2024); and (4) French green (lot# 24-040-WHQ-010) lentils were obtained from Washington state producers via www.bobsredmill.com (accessed on 10 April 2024). Notably, red and yellow lentils are commonly sold as split and dehulled, while green, brown, French green and black lentils are typically sold as whole with husk. In accordance with these commercial practices for human food consumption, this report utilized split, dehulled red and yellow lentils, while the other four types were used whole. Information regarding the agricultural origin (soil types, microclimates, and fertilizer regimes) and post-harvest storage times of these commercial lentil samples was unavailable. All lentils were purchased through Amazon.com in 2024 and immediately stored in a −20 °C freezer.

### 2.2. Lentil Cooking and Sample Pulverization

Cooking processes were performed according to USDA cooking instructions (https://wicworks.fns.usda.gov/topic/what-do-i-do-with/lentils, accessed on 6 May 2024) with minor modifications to achieve desired lentil firmness. Lentils were cooked on a Fisher Brand Isotemp hot plate (Cat# SP88850200) in an aluminum-foil-covered 600 mL beaker rinsed with element-free water. A total of 150 mL of element-free water was added to 75 g of dry red or yellow lentils, brought to a boil, and simmered for 10 min. Similarly, 190 mL of element-free water was added to 75 g of dry green, brown, French green, or black lentils; brought to a boil; and simmered for 30, 25, 25, and 20 min, respectively. The lentils and water were heated to 100 °C until boiling was achieved. Subsequently, the temperature of the sample mixture was reduced to ~90 °C to maintain a simmer. Cooking time started when boiling was initiated. While cooking, the lentils were swirled by hand to mix them every 5 min. Lentils were considered the desired firmness once several lentils could be smashed against the roof of the mouth with a testers’ tongue, without grittiness. After cooking, lentils and any residual cooking water were retained and moved to a plastic bag before being placed at −80 °C prior to lyophilization for 72 h (with a condenser temperature of −50 °C and a chamber vacuum pressure of 0.1 mbar). Thereafter, lentil samples were pulverized (500 μm particle size) using a Perten 3610 laboratory disk mill fitted with a Type 5 fine grinding plate (PerkinElmer, Shelton, CT, USA). Ground samples were collected in airtight bags and stored at −20 °C.

### 2.3. Chemical Analysis

#### 2.3.1. Protein, Fat, and Phenolic Content

A Dumas protein analysis (AOAC 968.06) was performed at Eurofins Analytics Laboratory (Madison, WI, USA) using a LECO CN828 Nitrogen Analyzer (LECO Corporation, St. Joseph, MI, USA). EDTA served as the calibration standard, and in-house QC-LCS samples were run throughout the procedure. Strict criteria for the atmospheric blank and EDTA samples had to be met during calibration before outside samples were analyzed. Because the Dumas method measures total nitrogen rather than protein directly, a crude protein conversion factor of 6.25 was utilized in the analysis. The fat content of lentil samples was measured using the CEM ORACLE Rapid NMR Fat Analyzer in tandem with the CEM SMART 6 moisture analyzer (CEM Corporation, Matthews, NC, USA). Approximately 3 g of pulverized lentil sample was weighed into a 6 mL sample tube prior to being verified on the SMART 6. Sample weights were verified, heated, and dried on glass fiber sample pads (CEM P/N 200150) on the SMART 6 to determine percent moisture and percent solids. Following moisture analysis, samples on sample pads were quickly wrapped in Trac Film (CEM P/N 159875) followed by transfer to the ORACLE, and the fat content was determined by the “dried beans” NMR method. The total phenolic content of a pulverized dry lentil sample (45 mg) was determined using a total phenol assay kit (Cat# NBP3-25868, Novus Biologicals, LLC, Centennial, CO, USA). Briefly, 45 mg of a pulverized lentil sample was mixed with 1 mL of 60% alcohol. The mixture was homogenized using a Tissuelyser for 60 s and centrifuged at 10,000× *g* for 10 min at room temperature. Following centrifugation, the supernatant was collected for detection at 760 nm using a microplate reader (Thermo Fisher Scientific, Waltham, MA, USA).

#### 2.3.2. Dietary Fiber and Profile

The total dietary fiber, soluble dietary fiber, and insoluble fiber of pulverized dry lentil samples were determined using an enzymatic–gravimetric (AOAC 991.43) method at Eurofins Analytics Laboratory (Madison, WI, USA). Similarly, the resistant starch content of the samples was determined using a standardized (AOAC 2002.02) method for measuring resistant starch (RS) at Eurofins Analytics Laboratory (Madison, WI, USA).

#### 2.3.3. Mineral Profile

A trace element analysis of lentil samples was performed using inductively coupled plasma mass spectroscopy (ICP-MS) on a NexION 5000 instrument (Perkin Elmer, Shelton, CT, USA). Prior to analysis, 200 mg dry weight (DW) aliquots of pulverized lentils, NIST 3234 (Soy Flour), and NIST 1567a (Wheat Flour) were transferred into high-purity quartz vessels (Blade, CEM Corporation, Matthews, NC, USA). An environmentally friendly digestion protocol was applied, consisting of 1 mL Optima^TM^-Grade HNO_3_ (Fisher Scientific, Hampton, NH, USA) and 4 mL Milli-Q^®^ deionized water (18 MΩ cm^−1^) in a CEM Blade microware system (with maximum temperature, 220 °C; pressure, 700 psi; ramp time, 5 min, and hold time 5 min). Following digestion, all resulting solutions were completely clear and free of particulate matter. The operational parameters for digestion and ICP-MS were adapted from previously described methods [13]. Element concentrations were determined via regression analysis using single-element calibration standards (AccuStandard, Inc., New Haven, CT, USA), with certified reference material recoveries yielding between 75% and 135%. Specifically, the % recovery rates for each element for both NIST SOY and NIST WHEAT were (1) Ca, 98.7% and 103.1%; (2) Cu, 85.9% and 134.7%; (3) Fe, 91.9% and 126.1%; (4) Mg, 108.9% and 86.5%; (5) Mn, 74.9% and 81.9%; (6) P, 103.9% and 107.8%; (7) K, 99.7% and 102.9%; (8) Na, 92.8% and 106.9%; and (9) Zn, 94.2% and 114.4%, respectively. Rhodium was used as an internal standard throughout the procedure.

### 2.4. Water- and Oil-Holding Capacity

The pulverized dry lentil samples (0.1 g per sample, with particle size 500 μm) were hydrated and blended with 10 mL of deionized water and vegetable oil (14.3% saturated fat, 57.2% polyunsaturated fat, and 25.0% monounsaturated fat), respectively. The hydration and mixing processes were conducted at room temperature (22 °C) for 1 h. As previously described [14], the total weight of the mixture in the centrifuge tube was recorded. Following centrifugation at 10,000× *g* for 10 min, the supernatant was drawn off via pipette as to not disturb the pellet, and the combined weight of the pellet and tube was measured. All procedures were performed in four replicates, and WHC and OHC were calculated as follows: (1) WHC (g/g) = (m2 − m1)/m2, and (2) OHC (g/g) = (m2 − m1)/m2, respectively, where m2 and m1 represent the wet and dry sample weights, respectively.

### 2.5. Statistical Analysis

While each lentil type had a single lot number, except for green lentils, which had two, the lentil sample for each experimental repetition came from a different small bag. For each lentil (red, yellow, green, brown, French green or black) type, the cooking process and sample pulverization were independently repeated on four different occasions. While a two-way analysis of variance (ANOVA) is the conventional approach for analyzing factorial designs, our primary interest was to evaluate the effect of cooking within each individual lentil type rather than estimate an overall cooking effect averaged across lentil types. Compared to the two-way ANOVA, a one-way ANOVA is more conservative for discovering true differences among the lentil types or between the two cooking methods (raw and cooked) with a lower false discovery rate (FDR). This approach treats the 12 combinations (2 × 6 = 12 groups) as independent groups and avoids comparing the two cooking methods by pooling data from the six lentil types. Thus, the mean values of raw and cooked lentils were compared using a one-way analysis of variance (ANOVA) with a false discovery rate (FDR) correction at a 0.05 significance level. Tukey contrasts were used for post hoc comparisons. All the data were presented as means (n = 4) ± SDs and analyzed by using JMP18 software (SAS Institute, Inc., Gary, NC, USA).

## 3. Results

### 3.1. Effect of Cooking and Lentil Types on Mineral Profile

Lentils are a rich source of various minerals, including calcium (Ca), phosphorus (P), potassium (K), zinc (Zn), magnesium (Mg), manganese (Mn), iron (Fe), copper (Cu), and sodium (Na). However, the mineral profile varies depending on the cooking process and the lentil type. The mineral content across lentil types was as follows (Table 1): (1) Ca ranged from 27.1 mg/100 g to 87.3 mg/100 g, with cooked brown lentils containing the highest amount of Ca and raw red lentils containing the lowest; (2) Cu ranged from 0.6 mg/100 g to 0.8 mg/100 g, with raw black lentils having the highest Cu and raw/cooked French lentils the lowest; (3) P ranged from 343.9 mg/100 g to 452.6 mg/100 g, with raw yellow lentils having the highest P and raw green lentils the lowest; (4) K ranged from 897.8 mg/100 g to 1183.8 mg/100 g, with raw yellow lentils having the highest K and raw brown lentils the lowest; (5) Mg ranged from 80.8 mg/100 g to 126.8 mg/100 g, with cooked black lentils having the highest Mg and cooked red lentils the lowest; (6) Fe and Zn were found in relatively small quantities, ranging from 4.6 mg/100 g to 6.8 mg/100 g for Fe and 2.7 mg/100 g to 4.3 mg/100 g for Zn, with cooked black lentils containing the highest amount of Fe and Zn. Similarly, Mn and Na were present in trace amounts, ranging from 0.7 mg/100 g to 1.0 mg/100 g for Mn and 0.4 mg/100 g to 3.6 mg/100 g for Na.

### 3.2. Effect of Cooking Processes and Lentil Types on Protein, Fat, and Phenolic Content

**Figure 2:**
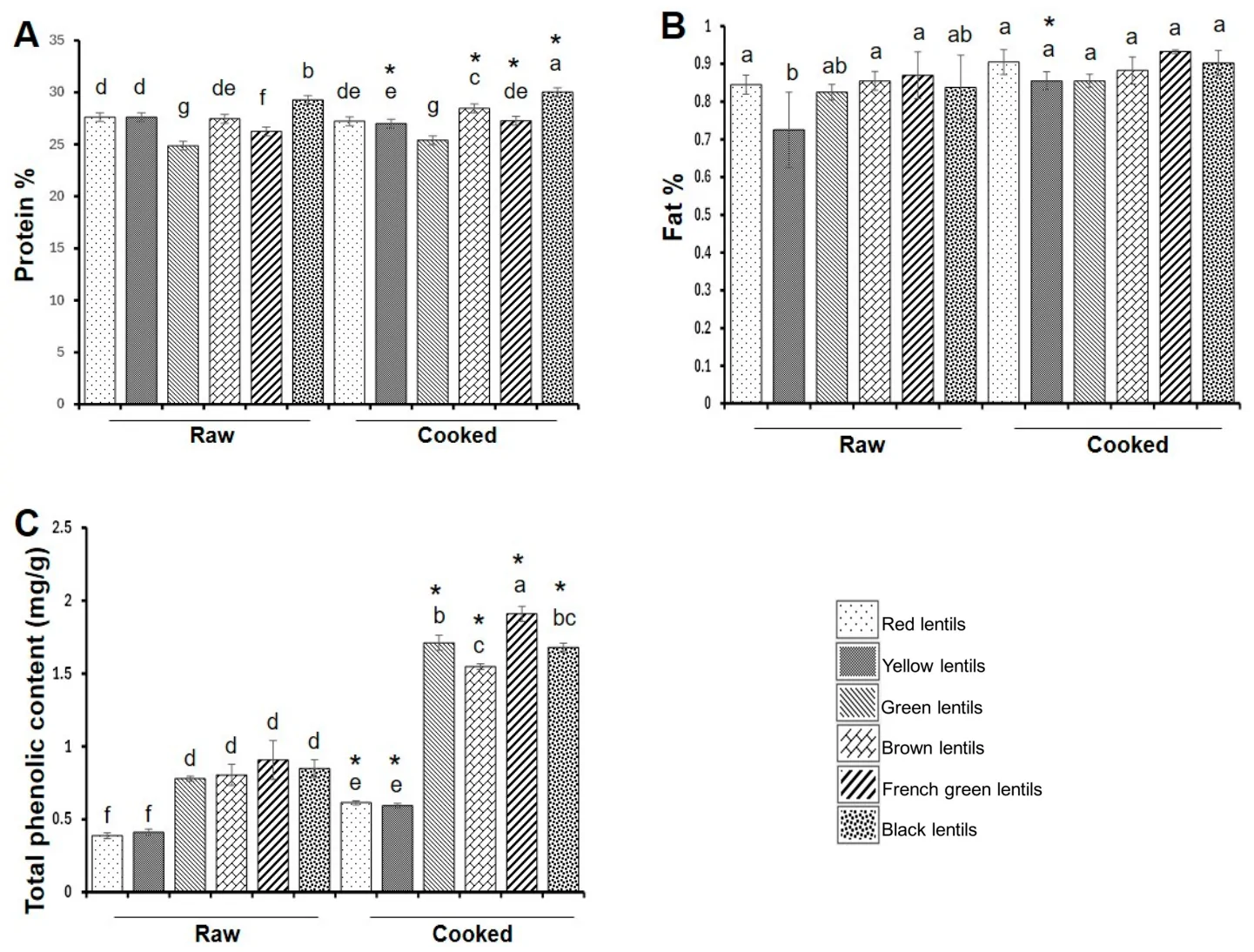
The effects of lentil type and the cooking process on (**A**) protein, (**B**) fat, and (**C**) total phenolic content. Values are means ± SD (n = 4 independent experiments). Values sharing a common letter are not statistically significant, while those with no letters in common differ significantly (*p* < 0.05). An asterisk * indicates a significant difference (*p* < 0.05) between a given cooked lentil type and its respective raw sample. The tabulated values are included in Supplementary Table S1.

The protein content of these six major lentil types (Figure 2A) ranged from 24.9% and 29.3% in their raw state and 25.4% and 30.0% when cooked. In both conditions, black lentils had the highest content, while green lentils had the lowest. Compared to their respective raw samples, the protein content of cooked yellow lentils decreased by 2.3%, whereas that of cooked French green, brown, and black lentils increased by 3.9%, 3.6%, and 2.5%, respectively.

The fat content of these six major lentil types (Figure 2B) was between 0.73% and 0.93% in both raw and cooked forms. Compared to the raw samples, the fat content of cooked yellow lentils increased by 17.9%, while no significant differences were found among the other five lentil types.

The total phenolic content of the six major lentil types (Figure 2C) ranged from 0.39 mg to 0.91 mg/g for raw samples and 0.59 mg to 1.91 mg/g for cooked samples. French green lentils had the highest phenolic content in both the raw and cooked conditions, while red and yellow lentils had the lowest in the raw and cooked states, respectively. Compared to their respective raw samples, the total phenolic content of cooked red, yellow, green, brown, French green, and black lentil samples increased by 58.3%, 44.1%, 119.7%, 92.5%, 110.5%, and 98.2%, respectively.

### 3.3. Effect of Cooking Processes and Lentil Types on Dietary Fiber

**Figure 3:**
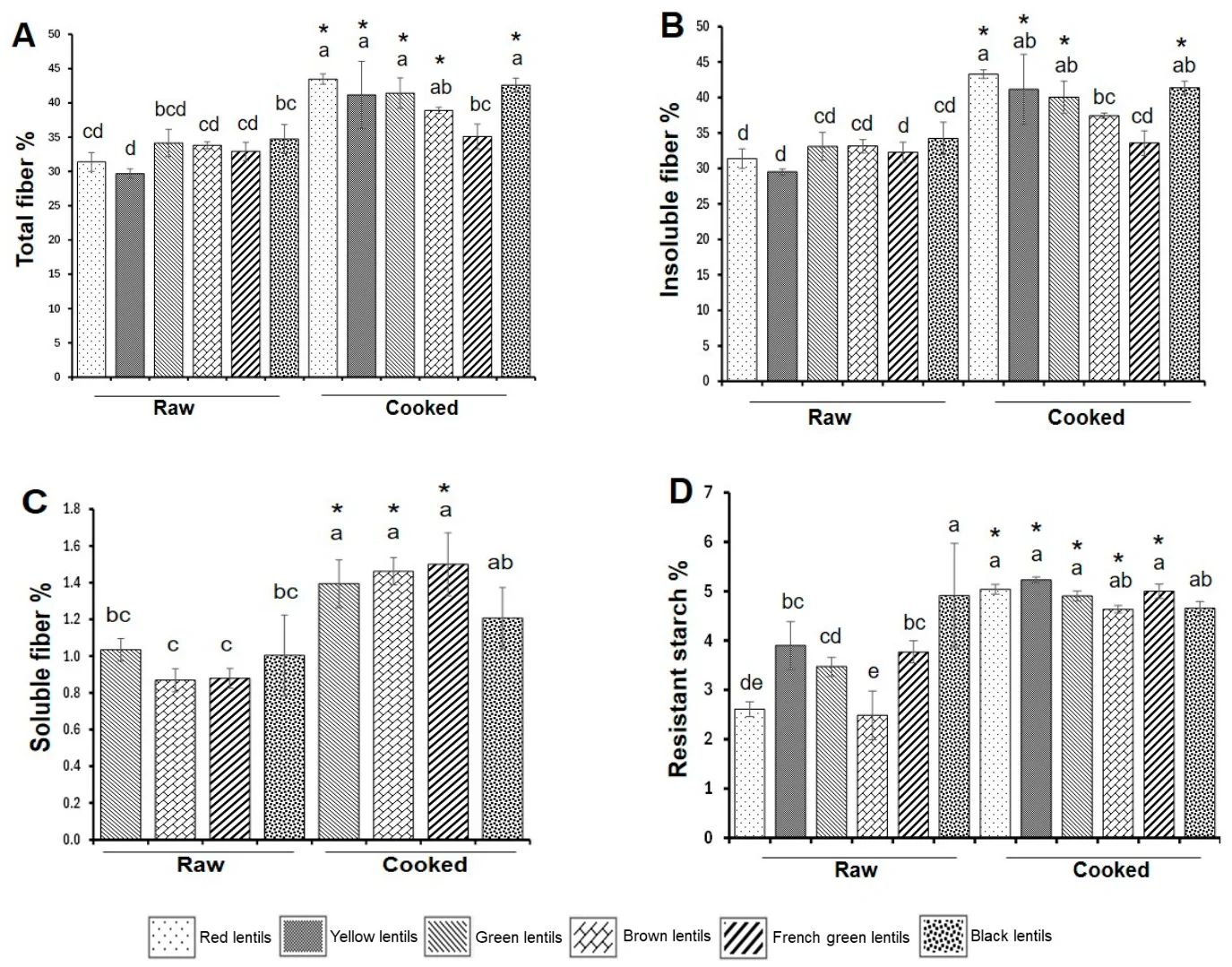
The effects of lentil type and the cooking process on (**A**) total, (**B**) insoluble, and (**C**) soluble fiber as well as (**D**) resistant starch content. Values are means ± SD (n = 4 independent experiments). Values sharing a common letter are not statistically significant, while those with no letters in common differ significantly (*p* < 0.05). An asterisk * indicates a significant difference (*p* < 0.05) between a given cooked lentil type and its respective raw sample. The tabulated values are included in Supplementary Table S2.

The total fiber content of the six major lentil types was as follows (Figure 3A): (1) in the raw state, levels ranged from 29.7% to 34.7%, with black lentils containing the highest percentage and yellow lentils the lowest, and (2) in the cooked state, levels increased to a range of 35.1% to 43.5%, with red and black lentils reaching the highest percentage and French green lentils the lowest. Compared to their respective raw samples, the levels in cooked brown, green, black, yellow, and red lentils increased by 15%, 21%, 23%, 39% and 39%, respectively.

The insoluble fiber content of the six major lentil types was as follows (Figure 3B): (1) in the raw state, levels ranged from 29.5% to 34.2%, with no significant differences found among the six types; (2) in the cooked state, levels ranged from 33.6% to 43.3%, with red lentils containing the highest percentages and French green lentils the lowest. Compared to their respective raw samples, the insoluble fiber content of cooked French green lentils remained unchanged. However, the fiber content in cooked brown, black, green, red, and yellow lentils increased by 13%, 21%, 21%, 38%, and 40%, respectively.

The soluble fiber content of green, brown, French green, and black lentils (Figure 3C) ranged from 0.87% to 1.03% for raw samples and from 1.21% to 1.50% for cooked samples, with no significant differences found among these four types in either their raw or cooked states. Compared to their respective raw samples, the soluble fiber content of green, brown, and French green lentils increased by 34%, 68%, and 71%, respectively. No detectable soluble fiber was found in raw or cooked red and yellow lentil samples.

The resistant starch content of these six major lentil types varied as follows (Figure 3D): (1) In the raw state, levels ranged from 2.5% to 4.9%, with black lentils containing the highest percentage and brown lentils the lowest; (2) when cooked, levels ranged from 4.6% to 5.2%, with no significant differences found among the six types. Compared to their respective raw samples, the content in cooked French green, yellow, green, brown, and red lentils increased by 33%, 34%, 41%, 87%, and 93%, respectively.

### 3.4. Effect of Cooking Processes and Lentil Types on Water- and Oil-Holding Capacity

**Figure 4:**
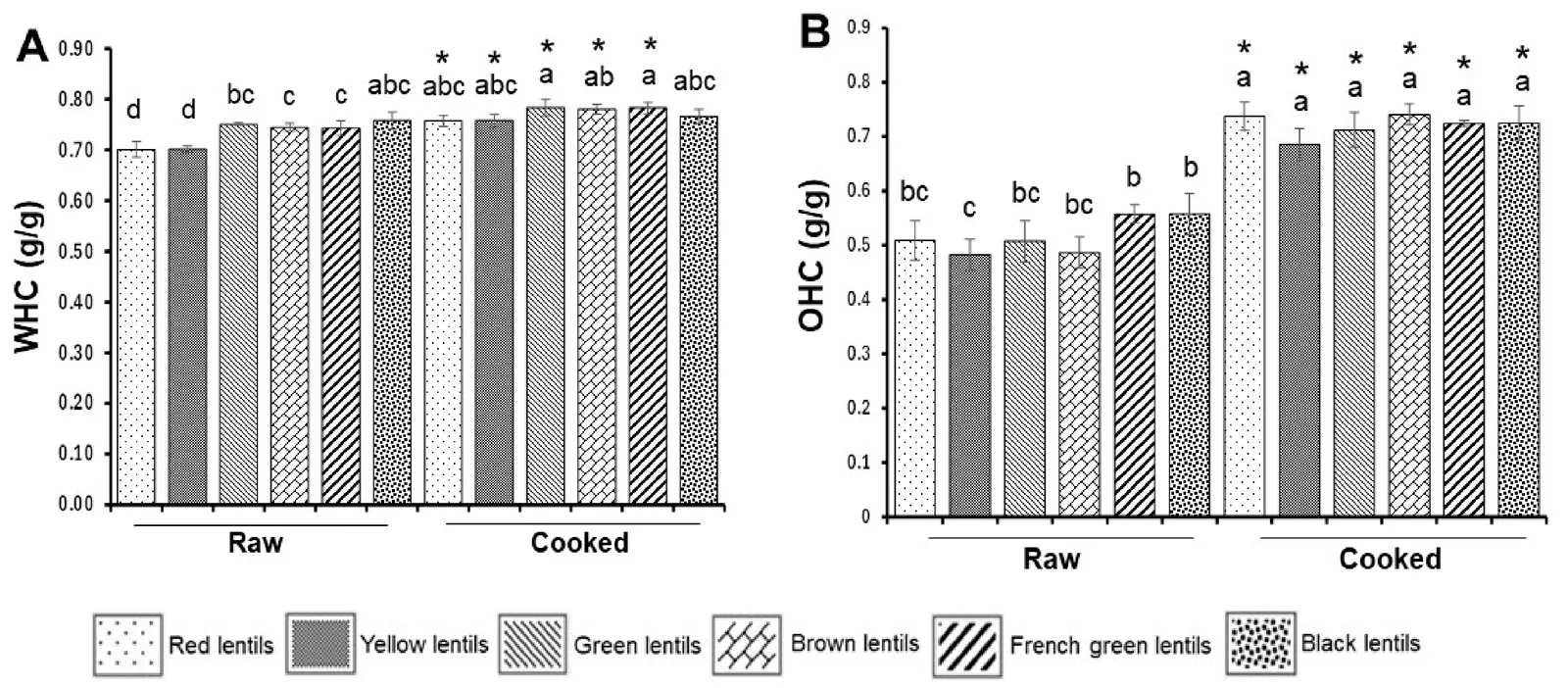
The effects of lentil type and the cooking process on (**A**) WHC and (**B**) OHC. Values are means ± SD (n = 4 independent experiments). Values sharing a common letter are not statistically significant, while those with no letters in common differ significantly (*p* < 0.05). An asterisk * indicates a significant difference (*p* < 0.05) between a given cooked lentil type and its respective raw sample. The tabulated values are included in Supplementary Table S3.

The water-holding capacity (WHC) of these six major lentil types was as follows (Figure 4A): (1) 0.70 to 0.76 g/g in the raw state, where black lentils had the highest values and red lentils the lowest, and (2) 0.76 to 0.78 g/g when cooked, with no significant differences found among the six types. Compared to their respective raw samples, the WHC of cooked red, yellow, green, brown, and French green lentils increased by 8.1%, 8.0%, 4.4%, 4.7%, and 5.4%, respectively.

The oil-holding capacity (OHC) of these six major lentil types (Figure 4B) ranged from 0.48 to 0.56 g/g in the raw condition; black lentils exhibited the highest OHC, while yellow lentils had the lowest. After cooking, the values increased to between 0.69 and 0.74 g/g, with no significant differences found among these six types. Compared to their respective raw samples, the OHC of cooked red, yellow, green, brown, French green and black lentils increased by 45.0%, 42.0%, 40.3%, 52.1%, 29.9% and 30.0%, respectively.

### 3.5. Impact of Resistant Starch and Dietary Fiber on WHC, OHC, and Other Nutrients

**Figure 5:**
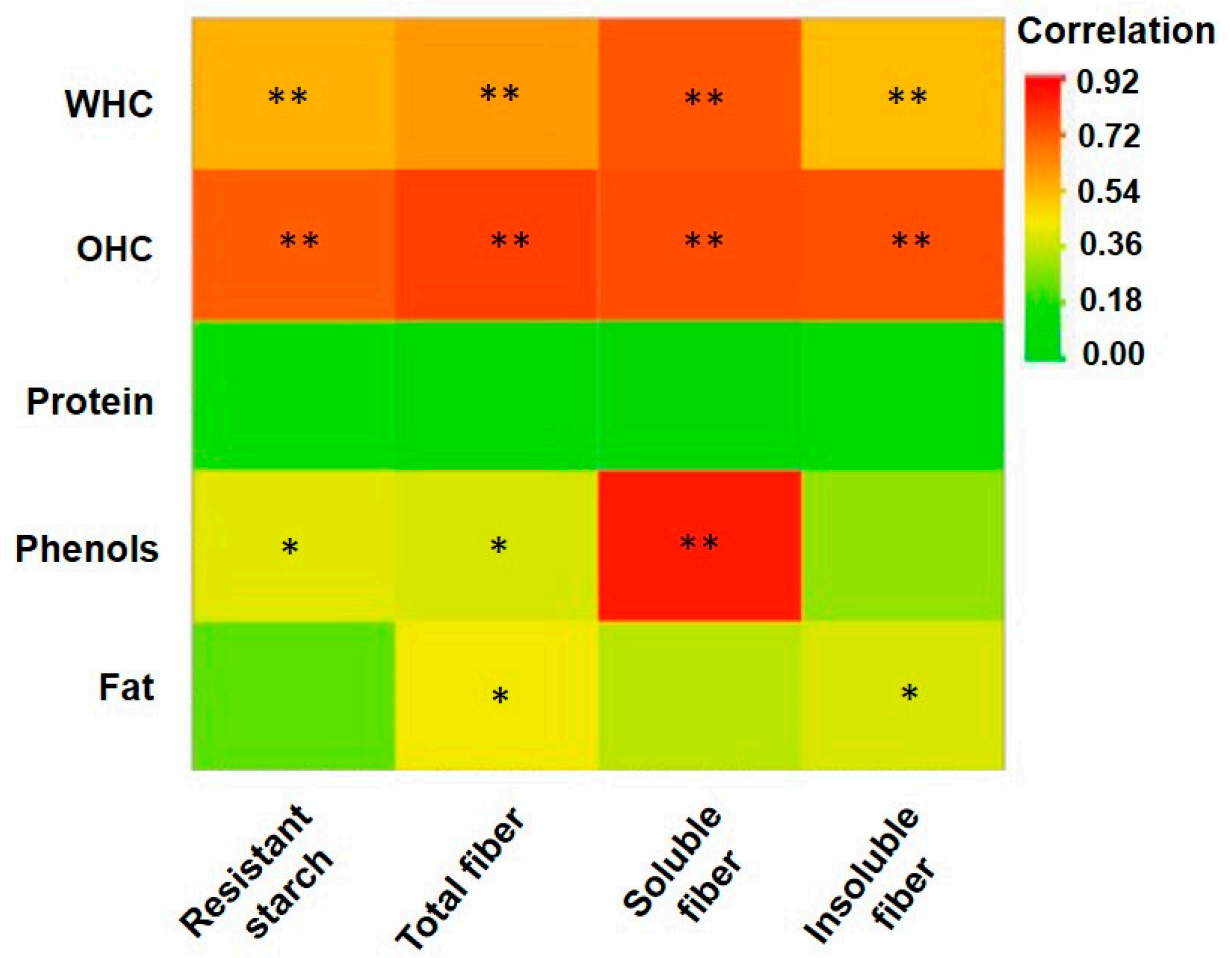
The correlation between (1) resistant starch/dietary fiber and (2) WHC, OHC and fiber-associated nutrients (protein, fat, total phenolic content). To focus on the impact of resistant starch and dietary fiber on WHC, OHC, protein, fat, and phenols, neither the lentil type nor the cooking factor should influence this correlation analysis. The heatmap color denotes the correlation coefficient r value. * *p* < 0.05 and ** *p* < 0.001 stand for a significant correlation adjusted by the FDR (n ≥ 30) from 4 independent experiments. The tabulated values are included in Supplementary Table S4.

To understand the impact of resistant starch and dietary fiber (soluble and insoluble) on WHC, OHC, protein, fat, and phytochemicals (e.g., total phenols), we analyzed their Pearson correlations (Figure 5). Resistant starch, total fiber, and soluble fiber were positively associated with WHC, OHC, and total phenolic content. Similarly, insoluble fiber showed positive associations with WHC and OHC, while fat content was positively associated with total and insoluble fiber.

## 4. Discussion

Lentils are exceptionally nutrient-dense, offering high protein, fiber, minerals and bioactive phytochemicals across all types. While they share a similar nutritional profile, specific types vary in texture and nutrition content. The main objective of this study was to assess the effects of lentil types and cooking processes on nutritional composition and physical properties such as OHC.

### 4.1. The Mineral Profile and Protein and Fat Content Vary Across Lentil Types

Specific mineral content fluctuates among different lentil types (Table 1): (1) P is an essential mineral with a recommended dietary allowance (RDA) of 700 mg/d for an American adult [15,16]; 16 to 20 g of dry lentils provides 10% of this daily allowance. (2) Fe is a key component of various proteins, including enzymes and hemoglobin, and the RDA for an American adult man and woman is 8 mg/day and 18 mg/day, respectively [17]. A daily consumption of 12 to 17 g of dry lentils for men and 27 to 37 g for women provides 10% of the daily Fe allowance. (3) K is an essential electrolyte that maintains fluid balance. The adequate intake (AI) is 3400 mg/day for men and 2600 mg/day for women [18]. Thus, a daily consumption of 29 to 38 g of dry lentils for men and 22 to 29 g for women provides 10% of the daily AI. (4) Cu is an essential component of metalloenzymes that function as oxidases. The RDA for Cu is 0.9 mg/day [17], and 11 to 14 g of dry lentils provides 10% of this allowance. (5) Mn is involved in bond formation as well as amino acid, lipid, and carbohydrate metabolism. While there is insufficient data to set an RDA, an AI for adult men and women is 2.3 and 1.8 mg/day, respectively [17]. Therefore, a daily consumption of 22 to 32 g of dry lentils for men and 17 to 25 g for women provides 10% of the daily AI (Table 1). (6) Mg is a cofactor in more than 300 enzyme systems. The RDA for adult men and women is 420 and 320 mg/day, respectively [19]. Consequently, a daily consumption of 33 to 52 g of dry lentils for men and 25 to 40 g for women provides 10% of the RDA for Mg (Table 1). (7) Zn is a component of various enzymes essential for the maintenance and regulation of gene expression, and the RDA for men and women is 11 and 8 mg/day, respectively [17]. Therefore, 26 to 41 g and 19 to 30 g of dry lentils provide 10% of the RDA of Zn for men and women, respectively. Collectively, at 50 g (about 1/4 cup), dry lentils constitute a practical and healthy daily serving, providing rich amounts of P, Fe, K, Cu, Mn, Mg, and Zn, though they are low in Ca and Na. Daily mineral intake is heavily influenced by lentil types.

The overall protein content of the cooked brown, French green, and black lentils was slightly higher than that of the raw lentils (Figure 2A). Consistent with previous data [20], the protein content of black lentils was the highest (~ 30%) among these six major lentil types. However, the observed change in the protein content of cooked versus raw lentils (Figure 2A) was unexpected; cooking is generally assumed to only affect protein structure rather than its total content when the cooking liquid is included. Thus, this observation suggests several potential contributing factors. First, the Dumas protein analytical method requires a step of combusting a sample to a high temperature [21]. Second, the physical forms of lentils were different: in contrast to the dehulled red and yellow lentils, the other four lentils were whole with husks. Third, the cooking process was a boiling procedure that retained the cooking liquid. Because the fibrous lentil husk may cause incomplete combustion during the Dumas protein analysis, the cooking process likely breaks down this complex fiber, which may improve combustion efficiency [22]. This may explain why the protein content ratios of cooked vs. raw lentils were increased in French green, brown and black (which had husks) but not red and yellow lentils. Conversely, the protein content ratio of cooked vs. raw lentils was decreased in dehulled yellow lentils. This observation suggests that the husk may prevent the loss of non-protein nitrogen (small molecules) species via certain gaseous forms of water during the boiling process, as the Dumas method measures total nitrogen, including non-protein nitrogen such as nitrates and nitrites. Similarly, while the overall fat content was low (≤ 1%) and comparable across the six major lentil types, an increase in the fat content of cooked versus raw yellow lentils was detected (Figure 2B). This observation suggests that cooking substantially alters the cellular matrix of yellow lentils, making bound lipids more accessible and resulting in a higher detectable fat content.

### 4.2. Cooking Alters Dietary Fiber, Phenolic Accessibility, WHC, and OHC

Lentil type may be a key factor driving lentil fiber content; for example, black lentils exhibited the highest dietary fiber content across the six major types (Figure 3). Cooking, specifically boiling, might also play a major role in lentil fiber content and subsequent WHC and OHC. Importantly, total, insoluble, and soluble fiber and resistant starch, WHC, and OHC increased by up to 39%, 40%, 71%, 93%, 8.1%, and 52.1% after boiling, respectively (Figure 3 and Figure 4). The boiling process may slightly decrease certain fiber fractions, such as that of hemicellulose. However, boiling could also break down starches and the plant cell wall matrix [22,23], which might lead to changes in plant cell component concentrations, such as an increase in the percentage of lignin and the release of phenolic compounds from the plant matrix [24,25]. Thermal energy from cooking breaks the chemical bonds that trap phenolic compounds inside the cell fibrous matrix [24,25]. This may explain an increase in the total detectable phenolic content in the cooked lentil samples (Figure 2C). Similarly, boiling denatures proteins and releases bound α-galactosides (oligosaccharides) from the lentil seed structure. Retaining the cooking water ensures that soluble proteins, phenols, and minerals are not lost, resulting in increased measurable fiber and phenolic content [26]. WHC is a key physicochemical property of insoluble dietary fiber capable of retaining water, encompassing bound water and physically trapped water under external centrifugal gravity [27]. Similarly, OHC is the ability of dietary fiber to physically or chemically absorb and retain significant quantities of oil [27]. Thus, these published data and proposed mechanisms may explain the observed increases in dietary fiber, phenols, WHC, and OHC after the cooking process (Figure 2C, Figure 3 and Figure 4).

### 4.3. Correlation Among WHC, OHC, Dietary Fiber, and Fiber-Associated Nutrients

Because the WHC and OHC of dietary fiber promote satiety and digestive health [27,28], they help prevent obesity. Therefore, we examined the relationships between these functional properties, dietary fiber, and fiber-associated nutrients. Dietary fiber can bind or entrap nutrients (e.g., proteins, fat, and phenols) through chemical bonds and physical barriers/encapsulation [27,28]. The correlation analysis (Figure 5) demonstrated that WHC, OHC, and total phenolic content were positively correlated with total, soluble, and insoluble fiber and resistant starch. These findings suggest several meaningful directions for future research: (1) It has been documented that bound polyphenols can be transported to the colon where they act as prebiotics for gut microbiota, producing beneficial short-chain fatty acids that reduce inflammation [29]. Does the positive correlation of resistant starch and fiber with phenols (in vitro) suggest that resistant starch and total and soluble fiber act as carriers for antioxidants (e.g., phenols), protecting these compounds from being degraded in the stomach in vivo? (2) At the in vivo level, dietary fiber swells in the stomach and intestines, which leads to prolonged satiety and helps control obesity [30,31]. Could the positive correlation of resistant starch, as well as total, soluble, and insoluble fiber, with WHC and OHC, in part, explain this in vivo observation? (3) Controlling rapid spikes in blood sugar and lipids is crucial for managing diabetes and cardiovascular health [32,33]. Does the positive correlation of total and insoluble fiber with fat suggest that total and insoluble fiber could play a greater role in binding and slowing down fat digestion when compared to resistant starch and soluble fiber? To address these important questions, future animal and human studies are needed before extrapolating these data to clinical application in the context of lentil consumption.

This report examined commercially available lentil types to compare their nutritional profiles, providing a quick snapshot of lentil-derived nutrient intake. However, this study has several limitations: (1) Each lentil type had only a single commercial lot number, except for green lentils, which had two. Including multiple lot numbers for each lentil type in upcoming projects may provide a comprehensive picture of general lentil nutrient consumption. (2) Starch retrogradation is a process where cooked and cooled starchy food realigns its starch molecules (amylose and amylopectin) into tight and crystalline structures, with an optimal cooling temperature at 4 °C. This chemical shift turns digestible starch into resistant starch [22,23]. Limited starch retrogradation may occur during the initial freezing stage when cooked samples are stored at an ultra-low temperature of −80 °C. This could partially explain the increase in resistant starch content in the cooked lentil samples (Figure 3D). However, other potential mechanisms for explaining the increase in resistant starch in cooked lentil samples in this report remain to be determined. (3) Only dehulled red and yellow lentils and unhulled green, brown, French green, and black lentils were commercially available. It will be of great interest to compare the nutritional composition of unhulled versus dehulled red and yellow lentils, which may explain the low values for soluble fiber and total phenols in the red and yellow lentil types in this report.

## 5. Conclusions

**Figure 6:**
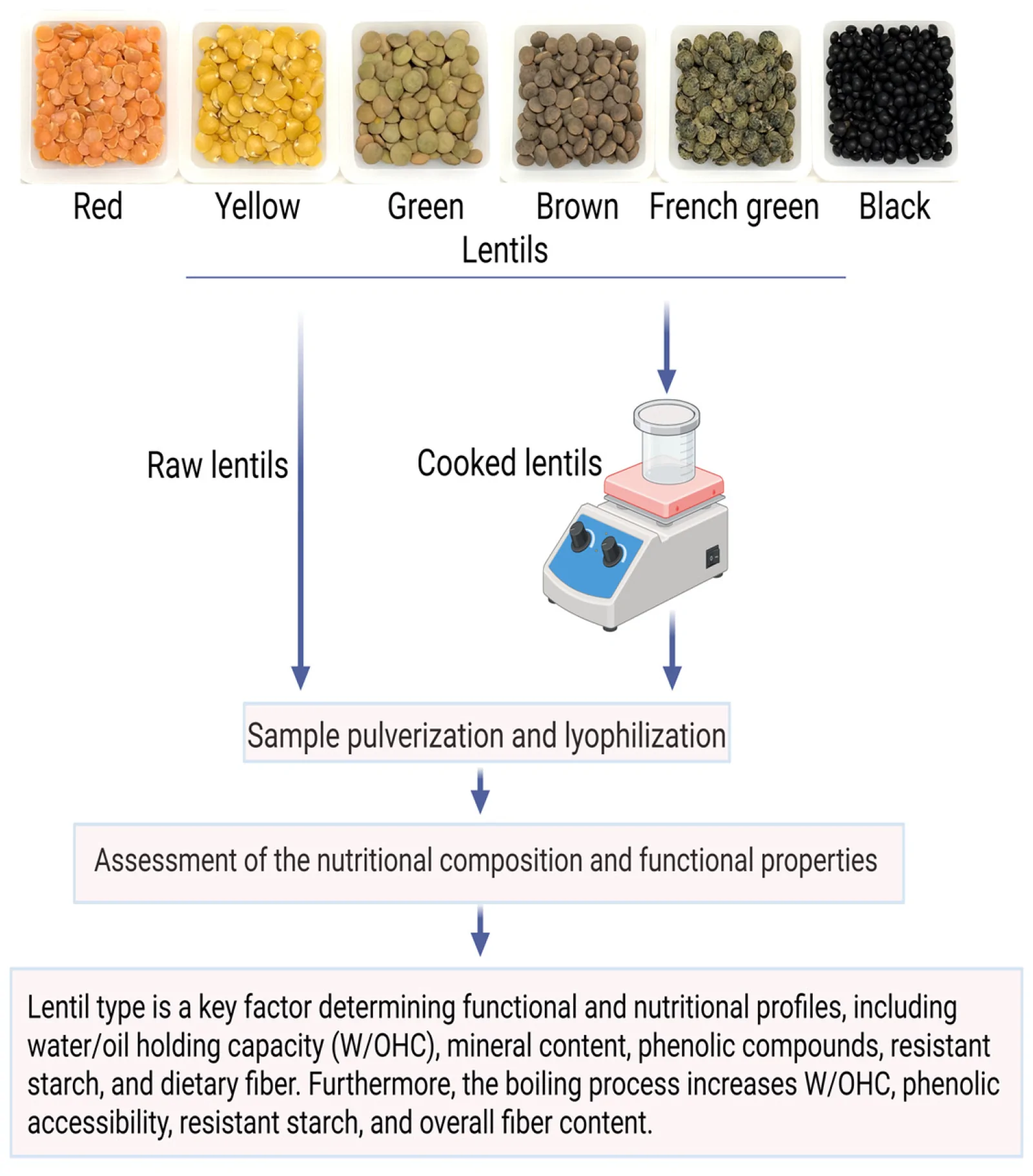
A schematic illustration of the effects of lentil type and cooking process on nutritional and functional properties (created with BioRender.com).

In summary, these data demonstrate that lentil type is a key determinant governing functional features and nutritional profiles, including WHC, OHC, mineral content, protein, fat, phenolic compounds, resistant starch, and dietary fiber (total, soluble, and insoluble). Furthermore, the cooking process enhances healthy eating indices, significantly increasing OHC, phenolic accessibility, resistant starch, and overall fiber content (Figure 6). These findings, particularly the high health indices of black lentils, offer valuable insights into the nutritional and health benefits of different lentil types.

## Figures and Tables

**Table 1:** The impact of lentil type and cooking processes on mineral composition.

Minerals	Treatment	Commercial Dry Lentil Type					
		Red	Yellow	Green	Brown	French Green	Black
Ca (mg/100 g)	Raw	27.1 ± 0.69 ^d^	30.8 ± 0.66 ^d^	71.8 ± 1.79 ^b^	84.6 ± 6.37 ^a^	63.1 ± 7.33 ^c^	59.8 ± 1.82 ^c^
	Cooked	27.6 ± 0.71 ^d^	30.8 ± 0.73 ^d^	73.0 ± 1.95 ^b^	87.3 ± 1.64 ^a^	67.0 ± 3.44 ^bc^	62.7 ± 1.62 ^c^
Cu (mg/100 g)	Raw	0.73 ± 0.01 ^bc^	0.78 ± 0.02 ^ab^	0.64 ± 0.01 ^ef^	0.72 ± 0.01 ^bcd^	0.63 ± 0.02 ^f^	0.82 ± 0.07 ^a^
	Cooked	0.67 ± 0.02 ^cdef^	0.76 ± 0.04 ^ab^	0.66 ± 0.02 ^def^	0.71 ± 0.02 ^bcde^	0.63 ± 0.04 ^f^	0.81 ± 0.01 ^a^
Fe (mg/100 g)	Raw	5.0 ± 0.23 ^de^	6.0 ± 0.56 ^b^	5.7 ± 0.20 ^bcd^	5.8 ± 0.40 ^bc^	5.7 ± 0.26 ^bcd^	6.8 ± 0.45 ^a^
	Cooked	4.6 ± 0.20 ^e^	* 5.1 ± 0.08 ^cde^	5.4 ± 0.31 ^bcde^	5.99 ± 0.38 ^b^	5.8 ± 0.34 ^bc^	6.8 ± 0.29 ^a^
Mg (mg/100 g)	Raw	84.8 ± 3.90 ^b^	110.9 ± 3.35 ^a^	120.5 ± 2.94 ^a^	114.2 ± 5.68 ^a^	112.3 ± 11.53 ^a^	117.9 ± 5.93 ^a^
	Cooked	80.8 ± 4.57 ^b^	* 94.7 ± 4.77 ^b^	116.7 ± 3.16 ^a^	111.3 ± 6.55 ^a^	117.3 ± 12.82 ^a^	126.8 ± 2.92 ^a^
Mn (mg/100 g)	Raw	0.88 ± 0.03 ^cd^	1.04 ± 0.06 ^a^	0.94 ± 0.03 ^bc^	0.86 ± 0.01 ^d^	0.95 ± 0.02 ^bc^	0.88 ± 0.04 ^cd^
	Cooked	* 0.73 ± 0.02 ^e^	* 0.90 ± 0.02 ^cd^	0.95 ± 0.02 ^bc^	0.92 ± 0.03 ^bcd^	* 1.04 ± 0.06 ^a^	* 1.00 ± 0.02 ^ab^
P (mg/100 g)	Raw	377.4 ± 5.9 ^cd^	452.6 ± 7.8 ^a^	343.9 ± 10.4 ^e^	361.4 ± 9.0 ^de^	375.6 ± 4.4 ^cde^	397.0 ± 26.9 ^bc^
	Cooked	350.9 ± 7.8 ^de^	427.6 ± 12.1 ^ab^	344.8 ± 11.5 ^e^	369.3 ± 13.2 ^cde^	363.0 ± 20.5 ^de^	* 419.4 ± 8.5 ^de^
K (mg/100 g)	Raw	1040.5 ± 23.4 ^bc^	1183.8 ± 65.3 ^a^	999.4 ± 33.2 ^cd^	897.8 ± 22.5 ^e^	922.6 ± 28.0 ^de^	935.9 ± 46.3 ^de^
	Cooked	1001.5 ± 22.1 ^cd^	1117.6 ± 43.4 ^ab^	1052.8 ± 35.6 ^bc^	990.6 ± 27.2 ^cde^	937.7 ± 70.6 ^de^	990.6 ± 14.9 ^cde^
Na (mg/100 g)	Raw	1.04 ± 0.09 ^b^	0.44 ± 0.02 ^c^	0.57 ± 0.03 ^bc^	0.39 ± 0.03 ^c^	3.55 ± 0.63 ^a^	0.51 ± 0.04 ^bc^
	Cooked	1.04 ± 0.11 ^b^	0.46 ± 0.07 ^c^	0.52 ± 0.05 ^bc^	0.42 ± 0.07 ^c^	3.67 ± 0.45 ^a^	0.48 ± 0.02 ^bc^
Zn (mg/100 g)	Raw	3.4 ± 0.13 ^de^	4.3 ± 0.17 ^a^	3.5 ± 0.10 ^de^	2.7 ± 0.04 ^f^	3.2 ± 0.09 ^e^	3.8 ± 0.28 ^bcd^
	Cooked	3.3 ± 0.11 ^e^	4.0 ± 0.08 ^abc^	3.6 ± 0.17 ^cde^	2.9 ± 0.25 ^f^	3.4 ± 0.24 ^e^	4.1 ± 0.06 ^ab^

Values are means ± SDs (*n* = 4 independent experiments). For a given mineral element in both raw and cooked forms, values sharing a common letter are not statistically significant, while those with no letters in common differ significantly (*p* < 0.05) adjusted by the FDR. An asterisk * indicates a significant difference (*p* < 0.05) between a given cooked lentil type and its respective raw sample. All lyophilized samples of either pulverized (raw) or cooked lentils had about an 8% moisture content. Commercial dry lentils (red, yellow, brown, green, and black) are safely stored and traded at <13% moisture content. Therefore, with a <5% moisture difference between the lyophilized samples and commercial dry lentils, the mineral values of the lyophilized (pulverized) samples in Table 1 should be highly similar to those of commercial dry lentils.

## Data Availability

The original contributions presented in this study are included in the article/Supplementary Material. Further inquiries can be directed to the corresponding author.

## Supplementary Materials

The following supporting information can be downloaded at https://www.mdpi.com/article/10.3390/foods15162898/s1.

## Abbreviations

AI, adequate intake; Ca, calcium; Cu, copper; Fe, iron; K, potassium; Mn, manganese; Mg, magnesium; Na, sodium; OHC, oil-holding capacity; P, phosphorus; RDA, recommended dietary allowance; WHC, water-holding capacity; Zn, zinc.

## References

[1] Kamalakantha T., Zavela C., Johnson L.K., Caligiuri S.P., Drewnowski A., Conrad Z. (2026). Greater Intake of Pulses Is Associated with Lower Prevalence of Cardiometabolic Diseases among Adults in the United States: NHANES, 1999 to 2018. Curr. Dev. Nutr..

[2] Liberal Â., Almeida D., Fernandes Â., Pereira C., Ferreira I.C., Vivar-Quintana A.M., Barros L. (2024). Nutritional, chemical, and antioxidant screening of selected varieties of lentils (*Lens culinaris* spp.) from organic and conventional agriculture. J. Sci. Food Agric..

[3] Chang T., Yang T., Ren M., Li X., Fang X., Niu B., Yang H., Wang L., Chen X. (2025). Screening and Validation of Rhizobial Strains for Improved Lentil Growth. Microorganisms.

[4] Gan Y., Hamel C., O’Donovan J.T., Cutforth H., Zentner R.P., Campbell C.A., Niu Y., Poppy L. (2015). Diversifying crop rotations with pulses enhances system productivity. Sci. Rep..

[5] Ganesan K., Xu B. (2017). Polyphenol-Rich Lentils and Their Health Promoting Effects. Int. J. Mol. Sci..

[6] Chamberlin M.L., Wilson S.M.G., Gaston M.E., Kuo W.Y., Miles M.P. (2024). Twelve Weeks of Daily Lentil Consumption Improves Fasting Cholesterol and Postprandial Glucose and Inflammatory Responses-A Randomized Clinical Trial. Nutrients.

[7] Bresciani A., Emide D., Saitta F., Fessas D., Iametti S., Barbiroli A., Marti A. (2022). Impact of Thermal Treatment on the Starch-Protein Interplay in Red Lentils: Connecting Molecular Features and Rheological Properties. Molecules.

[8] Vashishth R., Semwal A.D., Naika M., Sharma G.K., Kumar R. (2021). Influence of cooking methods on antinutritional factors, oligosaccharides and protein quality of underutilized legume Macrotyloma uniflorum. Food Res. Int..

[9] Margier M., Georgé S., Hafnaoui N., Remond D., Nowicki M., Du Chaffaut L., Amiot M.J., Reboul E. (2018). Nutritional Composition and Bioactive Content of Legumes: Characterization of Pulses Frequently Consumed in France and Effect of the Cooking Method. Nutrients.

[10] Rondanelli M., Daglia M., Meneghini S., Di Lorenzo A., Peroni G., Faliva M.A., Perna S. (2017). Nutritional advantages of sous-vide cooking compared to boiling on cereals and legumes: Determination of ashes and metals content in ready-to-eat products. Food Sci. Nutr..

[11] Perera D., Devkota L., Garnier G., Panozzo J., Dhital S. (2023). Hard-to-cook phenomenon in common legumes: Chemistry, mechanisms and utilisation. Food Chem..

[12] Vargha S., Igual M., Miraballes M., Gámbaro A., García-Segovia P., Martínez-Monzó J. (2024). Influence of Cooking Technique on Bioaccessibility of Bioactive Compounds in Vegetable Lentil Soup. Foods.

[13] Bussan D.D., Nielsen F.H., Douvris C., Kelzenberg B., Grimestad A., Cao J.J. (2024). An Environmentally Compatible and Less Costly (Greener) Microwave Digestion Method of Bone Samples Using Dilute Nitric Acid for Analysis by ICP-MS. Molecules.

[14] Hou C., Zhang Y., Chen J., Hu J., Yang C., Chen F., Zhu T., Xin Y., Geng X. (2025). Optimization of Solid-State Fermentation Process for Dietary Fiber in Flaxseed Meal and Analysis of Its Microstructure and Functional Properties. Foods.

[15] Institute of Medicine, Standing Committee on the Scientific Evaluation of Dietary Reference Intakes (1997). The National Academies Collection: Reports funded by National Institutes of Health. Dietary Reference Intakes for Calcium, Phosphorus, Magnesium, Vitamin D, and Fluoride.

[16] Fulgoni K., Fulgoni V.L. (2021). Trends in Total, Added, and Natural Phosphorus Intake in Adult Americans, NHANES 1988-1994 to NHANES 2015-2016. Nutrients.

[17] Institute of Medicine, Panel on Micronutrients (2001). Dietary Reference Intakes for Vitamin A, Vitamin K, Arsenic, Boron, Chromium, Copper, Iodine, Iron, Manganese, Molybdenum, Nickel, Silicon, Vanadium, and Zinc.

[18] Hoy M.K., Goldman J.D., Moshfegh A. (2010). Potassium Intake of the U.S. Population: What We Eat in America, NHANES 2017-2018. FSRG Dietary Data Briefs.

[19] Fatima G., Dzupina A., Alhmadi H.B., Magomedova A., Siddiqui Z., Mehdi A., Hadi N. (2024). Magnesium Matters: A Comprehensive Review of Its Vital Role in Health and Diseases. Cureus.

[20] Piergiovanni A.R. (2021). Nutritional Characteristics of Black Lentil from Soleto: A Single-Flower Vetch Landrace of Apulia Region (Southern Italy). Foods.

[21] Muñoz-Huerta R.F., Guevara-Gonzalez R.G., Contreras-Medina L.M., Torres-Pacheco I., Prado-Olivarez J., Ocampo-Velazquez R.V. (2013). A review of methods for sensing the nitrogen status in plants: Advantages, disadvantages and recent advances. Sensors.

[22] Vasishtha H., Srivastava R.P. (2013). Effect of soaking and cooking on dietary fibre components of different type of chickpea genotypes. J. Food Sci. Technol..

[23] Yadav B.S., Sharma A., Yadav R.B. (2010). Resistant starch content of conventionally boiled and pressure-cooked cereals, legumes and tubers. J. Food Sci. Technol..

[24] Mustafa A.M., Abouelenein D., Acquaticci L., Alessandroni L., Angeloni S., Borsetta G., Caprioli G., Nzekoue F.K., Sagratini G., Vittori S. (2022). Polyphenols, Saponins and Phytosterols in Lentils and Their Health Benefits: An Overview. Pharmaceuticals.

[25] Nowak K., Rohn S., Halagarda M. (2025). Impact of Cooking Techniques on the Dietary Fiber Profile in Selected Cruciferous Vegetables. Molecules.

[26] Amoah I., Ascione A., Muthanna F.M.S., Feraco A., Camajani E., Gorini S., Armani A., Caprio M., Lombardo M. (2023). Sustainable Strategies for Increasing Legume Consumption: Culinary and Educational Approaches. Foods.

[27] Liu T., Zhen X., Lei H., Li J., Wang Y., Gou D., Zhao J. (2024). Investigating the physicochemical characteristics and importance of insoluble dietary fiber extracted from legumes: An in-depth study on its biological functions. Food Chem. X.

[28] Ariyarathna P., Mizera P., Walkowiak J., Dziedzic K. (2025). Physicochemical and Functional Properties of Soluble and Insoluble Dietary Fibers in Whole Grains and Their Health Benefits. Foods.

[29] Awika J.M., Rose D.J., Simsek S. (2018). Complementary effects of cereal and pulse polyphenols and dietary fiber on chronic inflammation and gut health. Food Funct..

[30] Daley S.F., Shreenath A.P. (2026). The Role of Dietary Fiber in Health Promotion and Disease Prevention: A Practical Guide for Clinicians. StatPearls.

[31] Kabisch S., Hajir J., Sukhobaevskaia V., Weickert M.O., Pfeiffer A.F.H. (2025). Impact of Dietary Fiber on Inflammation in Humans. Int. J. Mol. Sci..

[32] Ben Jeddou K., Bouaziz F., Zouari-Ellouzi S., Chaari F., Ellouz-Chaabouni S., Ellouz-Ghorbel R., Nouri-Ellouz O. (2017). Improvement of texture and sensory properties of cakes by addition of potato peel powder with high level of dietary fiber and protein. Food Chem..

[33] Burton-Freeman B. (2000). Dietary fiber and energy regulation. J. Nutr..

